# Associated Factors and Standard Percentiles of Blood Pressure among the Adolescents of Jahrom City of Iran, 2014

**DOI:** 10.1155/2017/3804353

**Published:** 2017-01-16

**Authors:** Yaser Sarikhani, Seyed Taghi Heydari, Fatemeh Emamghorashi, Fatemeh Jafari, Reza Tabrizi, Saeed Karimpour, Ahmad Kalateh sadati, Maryam Akbari

**Affiliations:** ^1^Research Center for Social Determinants of Health, Jahrom University of Medical Sciences, Jahrom, Iran; ^2^Health Policy Research Center, Shiraz University of Medical Sciences, Shiraz, Iran; ^3^Student Research Committee, Jahrom University of Medical Sciences, Jahrom, Iran; ^4^Department of Sociology, Yazd University, Yazd, Iran

## Abstract

*Background*. High blood pressure in adults is directly correlated with increased risk of cardiovascular diseases. Hypertension in childhood and adolescence could be considered among the major causes of this problem in adults. This study aimed to investigate the factors associated with hypertension among the adolescents of Jahrom city in Iran and also standard percentiles of blood pressure were estimated for this group.* Methods*. In this community-based cross-sectional study 983 high school students from different areas of the city were included using a multistage random cluster sampling method in 2014. Blood pressure, weight, and height of each student measured using standard methods. Data were analyzed by statistical software SPSS 16.* Results*. In total, 498 male and 454 female students were included in this study. Average systolic blood pressure of students was 110.27 mmHg with a variation range of 80.6–151.3. Average diastolic blood pressure was 71.76 mmHg with the variation range of 49.3–105. Results of this study indicated that there was a significant relationship between gender, body mass index, and parental education level with systolic and diastolic blood pressure of the students (*P* < 0.05).* Conclusions*. Body mass index was one of the most important changeable factors associated with blood pressure in adolescents. Paying attention to this factor in adolescence could be effective in prevention of cardiovascular diseases in adulthood.

## 1. Introduction

Nowadays cardiovascular diseases are among the leading causes of death all around the world [1]. Among the different predisposing causes of these problems hypertension is considered as an important risk factor [2]. About 80 percent of global hypertension cases occur in developing countries. In Iran 50 percent of deaths due to cardiovascular diseases is related to hypertension [3].

Hypertension which usually occurs in adults is determined by different factors such as gender, weight, height, genetic, physiologic, lifestyle, diet, geographical conditions, and ethnicity [4, 5]. Also hypertension occurred in all age groups, but it is obvious that prevalence of hypertension in a population rises along with the increase of the age [6].

Systemic hypertension is an indication of an underlying pathophysiology in childhood and adolescence which if not controlled effectively could be resulted to the increased risk of myocardial and cerebral infarctions in adulthood [7]. Studies show that hypertension in early adulthood age has its root in adolescence [8].

Hypertension in children is often asymptomatic and does not receive any particular treatment. Hypertension pharmaceutical treatment usually starts in adulthood, whereas high blood pressure (BP) has left its considerable damaging effect on vascular system and other organs of the body from childhood which are often irreversible [9]. Many prospective studies indicated that children with sustainable high BP had higher risk of hypertension in adulthood [10]. Therefore hypertension screening in childhood and adolescence could result in recognition of people with early hypertension and those with higher risk of developing the problem [11].

Forasmuch BP in childhood and adolescence is affected by different factors such as age, gender, height, weight, ethnicity, nutrition, geographic zone, stage of sexual development, and even fatal growth pattern. BP level and its changes vary in different populations. Many studies have reported prevalence of hypertension among children and adolescence from 1 to 16.6 percent [12–14].

Studies done on children and adolescents have shown that different factors (such as gender, age, height, and body size) are associated with BP in these groups [15]. It should be considered that as these factors vary in different communities and since that children and adolescents are in a dynamic growth process, there are no specific standard criteria for analysis of BP pattern and also diagnosis and treatment of hypertension in these groups [16].

This study aimed to investigate factor associated with BP and also to determine its standard percentiles in adolescents of Jahrom city in southern Iran.

## 2. Methods

This cross-sectional community-based study was done in Jahrom city of Iran in 2014. Jahrom with 220000 inhabitants is one of the most populated cities of Fars province (the fourth province of the country in terms of area and population) which is located in southern Iran.

We used a multistage random cluster sampling method for sampling and accordingly 8 high schools (4 male and 4 female schools) were selected from different areas of the city as clusters. Different educational levels (4 classes) were considered as strata and then we selected samples from these strata randomly. Finally 983 students including 498 male and 454 female students were selected.

Necessary data were gathered from the entire participants who include age, gender, height, weight, systolic and diastolic BP, physical activity, and some demographic information. To measure the variables we used 6 public health technicians who were working under the supervision of a general physician.

Age of the participants retrieved from their registration documents in the schools. We used a digital stadiometer (Seca 274) for measurement of height and weight of the students. In order to reduce error of measurement, the stadiometer was calibrated with a standard weight every day after using it. Weight of participants measured with an accuracy of 0.1 Kg. In order to increase the accuracy of measurement participants were assessed with minimum possible cloths and also they were asked not to wear shoes and hat.

We used a digital sphygmomanometer (Omron M6 Comfort) to measure BP of which length of its cuff was at least 2.3 times more than arm circumference of participants. BP of students measured twice with an interval of 5 minutes in a sitting position. The average value of two measurements was considered as BP of participants. In order to increase reliability of the data, all of the BP measurements were done by one of the technicians using the same method. In all cases diaphragm of the stethoscope located on brachial artery, the cuff filled 30 to 40 mmHg more than expected systolic blood pressure (SBP), and then the air released by 3 mmHg in second. The appearance point of the first Korotkoff sounds was considered as SBP. In the cases that Korotkoff sounds were audible to 0 mmHg, the appearance point of forth Korotkoff sounds was considered as diastolic blood pressure (DBP). The sphygmomanometer and stethoscope were checked for likely error every day. Four team groups were trained to collect data by measurement height, weight, BP, and other variables.

In order to increase measurement accuracy some advices were recommended including the following: all participants got rest in a calm and quiet place at least 5 minutes before BP measurement; to reduce the likely stresses all participants were informed about the process; to avoid the effects of dilated urinary bladder all participant were asked to dispose their urine before measurement if needed; the participants were asked not to use caffeine and any adrenergic stimulant such as eye and nose drops and should not be involved in a hard physical activity or severe exercise 1 hour before measurement.

All of the participants were in healthy children situation, had not any known disease, and were not under any drug therapy plan. Suspected participants checked by the physician and cases with illnesses and comorbidities (including genetic and congenital disorders, cardiovascular diseases, physical disabilities, Asthma, and chronic kidney disease) were not included in sampling.

This study was approved by the ethical committee of Jahrom University of Medical Sciences. All of the cases were informed about the study and participated with the consent.

### 2.1. Statistical Analyses

Data were entered into the statistical software for windows (SPSS 16). The descriptive variables such as mean and standard deviations were used. After checking normality assumption by Kolmogorov-Smirnov test, independent *t*-test was used to compare SBP and DBP with gender. One-way analysis of variance (ANOVA) was performed for finding out significance difference among mean of SBP and DBP with body mass index, father and mother education levels, regular physical exercise, and participants' education levels. *P* value less than 0.05 was considered statistically significant.

Smoothed gender specific reference plots showing 3th, 10th, 15th, 25th, 50th, 75th, 85th, 90th, and 97th percentiles were derived using LMS method (LMS Chartmaker Pro version 2.4, 2008; by Dr. Huiqi Pan and Dr. Tim Cole) [17]. SBP and DBP were summarized by three smooth curves plotted against age, representing the median (*M*), coefficient of variation (*S*), and skewness (*L*) of the measurement distribution [18]. Models were checked for goodness of fit using the detrended *Q*-*Q* plot, *Q* tests, and worm plots [19]. The LMS method was found to be appropriate to use for this data as the measure of skewness of the data was 1.2 with a standard error of 0.07.

### 2.2. Results

In this study 983 students from 8 high schools of Jahrom city were included. 50.56% (497) of the participants were male with the mean ± SD age of 16.3 ± 1.1 (range: 14.2–20.7) and 49.44% (486) of them were female with the mean ± SD age of 15.8 ± 1.2 (range: 13.8–19.8). Mean ± SD SBP of the students was 110.27 ± 11.44 mmHg (range: 80.60–151.3) and the mean ± SD DBP was 71.76 ± 8.61 mmHg (range: 49.3–105). Also, mean ± SD weight and height were 59.33 ± 13.3 (range 33.7–122.9) and 165.2 ± 7.8 (rang 138–189), respectively.

Averages of SBP and DBP of the students in terms of gender, body mass index, parents' education level, physical activity, and student's education level are presented in Table 1.

**Figure 1:**
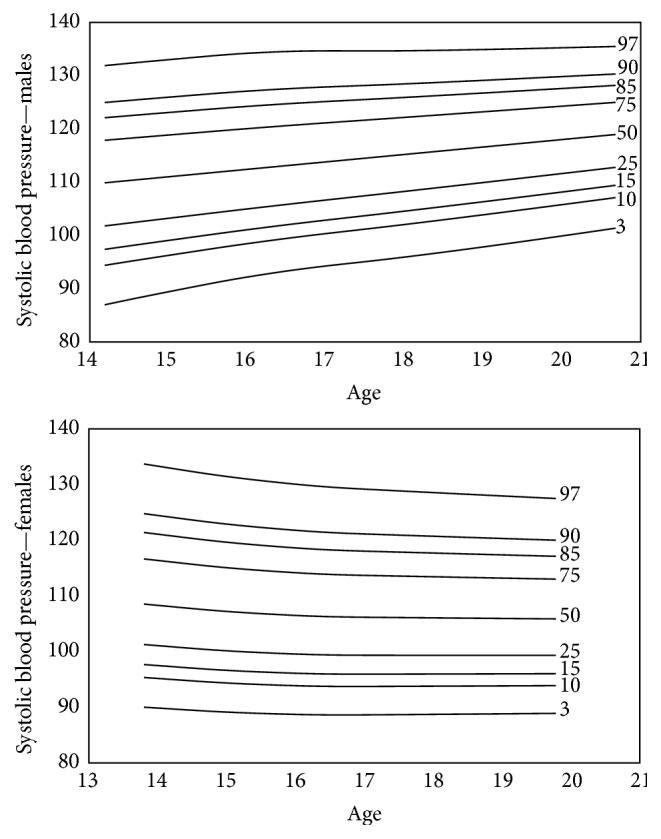
The smoothed standard percentiles of systolic blood pressure among 13–21-year-old adolescents of Jahrom city.

**Figure 2:**
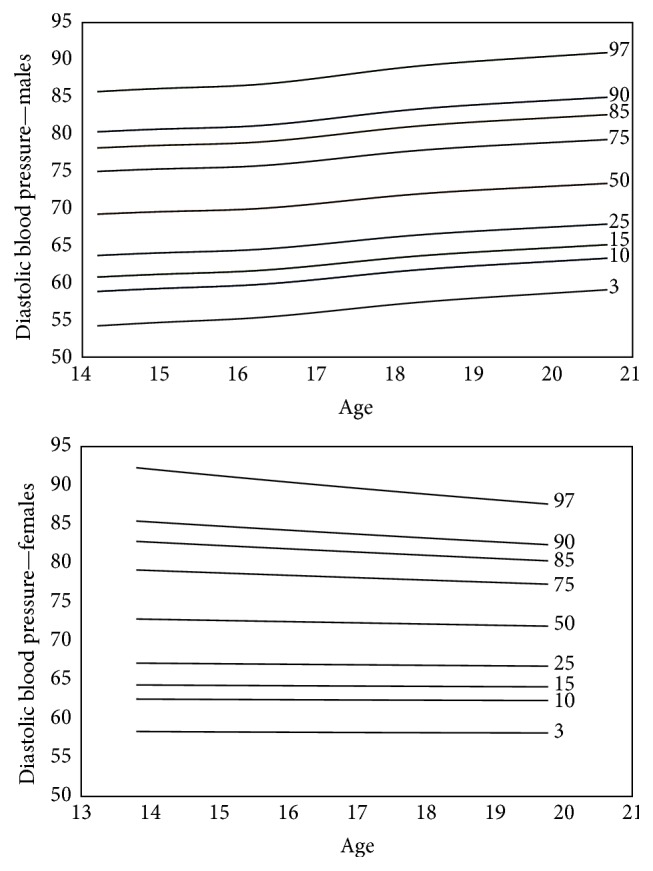
The smoothed standard percentiles of diastolic blood pressure among 13–21-year-old adolescents of Jahrom city.

After completion of the study the standard percentiles of BP were estimated for students of Jahrom city in both genders. The model was considered a good fit as per the shape of the worm plot; the *Q* statistic curves for *L*, *M*, and  *S* were within −2 and +2, and the detrended *Q*-*Q* plot indicated that the population was approximately normal. Figures 1 and 2 show the standard percentiles of BP in terms of gender and age.

## 3. Discussion

Hypertension in adults could result in various complications imposing financial and nonfinancial burdens to individuals and societies [20]. Hypertension in adolescents is considered as one of the important causes of this problem. Therefor monitoring of BP in childhood and adolescence could be helpful for early detection and prevention of hypertension in adults [21].

According to the results of this study average of SBP among the male students was 5.2 mmHg more than females, whereas the average of DBP among females was 2.5 mmHg more than males. Results of the studies that have evaluated the relationship between gender and BP are very different. Whereas many studies, such as Nielsen and Andersen in Denmark [22], Al-Sendi et al. in Bahrain [14], and Nader et al. [23], in Iran have suggested that average of BP among male adolescents was more than females, some other studies such as Omisore et al. in Nigeria [24] and Falah et al. [13] in Iran have indicated different findings.

On the other hand results of some studies such as de Rezende et al. in Brazil [10] and Ataei et al. in Iran [25] did not report a significant difference between average of BP among male and female adolescents. It seems that gender difference in average blood pressure could be result of different life styles, dietary habits, and sociodemographic factors in societies and also different study designs.

Many similar studies, in line with our findings, have confirmed a direct relationship between BMI and BP. Studies of Ribeiro et al. in Portugal [26], He et al. [27] in China, and Soleymani et al. [28] in Iran indicated that average of blood pressure significantly increased along with the body mass index. It could be noted that dietary habits and pattern of physical activity among the obese and overweight people are important factors associated with hypertension.

Findings of this study suggested that there was no significant correlation between regular physical exercise and average of SBP and DBP among adolescents. Similar studies which have been done in this area have found conflicting results. Study of Strazzullo et al. [29] in Australia revealed that there was an indirect relationship between regular physical exercise and hypertension among children and adolescents, while Corrêa Neto et al. [30] did not find a strong correlation between physical exercise and blood pressure among Brazilian children and adolescents. Given that physical activity in children and adolescents is not limited to regular sport activities, definite conclusion about the relationship between BP and regular physical activity is impossible and the correlation should be evaluated through the longitudinal studies.

This study showed that average of SBP and DBP of students was indirectly associated with the education level of parents, so that average of blood pressure was higher among the students whose parents had lower education levels. It is believed that parental education level is correlated with healthy dietary pattern and life style of students. Consumption of salt, sugar, and cooking oils which contain polyunsaturated fatty acids is higher among the families with lower health knowledge. This kind of nutrition pattern as well as lack of enough attention to regular appropriate physical activity could lead to hypertension in children and adolescents. Also it is assumed that some parents with lower educational level consider their children's overweight and obesity as a sign of health which will result in overnutrition of children.

Hypertension among children and adolescents that is always asymptomatic is a leading risk factor of cardiovascular diseases in adults. Therefor detection and control of high blood pressure in this period are considered as an appropriate preventive approach to reducing complications in adults.

Findings of this study indicated that different factors are associated with average of blood pressure in students. Body mass index is one of the main factors that is influenced by various determinants such as nutrition pattern, physical activity, and also heredity. Intervention strategies should focus on the most important factors that have been determined through these kinds of studies.

## Figures and Tables

**Table 1:** Average of systolic and diastolic blood pressure among Jahrom high school students in terms of demographic and anthropometric factors.

Characteristics		Blood pressure^*∗∗∗*^		*P* value
Gender	Male	SBP^*∗*^	112.8 ± 11.1	<0.001
	Female		107.6 ± 11.1	
	Total		110.27 ± 11.4	
	Male	DBP^*∗∗*^	70.5 ± 8.4	<0.001
	Female		73 ± 8.6	
	Total		71.76 ± 8.6	

Body mass index	<18.5	SBP	106.3 ± 10.9	<0.001
	18.5–25		110.3 ± 11.1	
	25–30		114.2 ± 10.4	
	>30		117.5 ± 13.3	
	<18.5	DBP	71.2 ± 8.3	<0.001
	18.5–25		70.9 ± 8.4	
	25–30		74 ± 8.4	
	>30		78 ± 9.3	

Father's education level	Illiterate	SBP	114.4 ± 9.4	0.01
	Under diploma		110.1 ± 11.6	
	Diploma		109.2 ± 11	
	University		111.3 ± 11.7	
	Illiterate	DBP	73 ± 8.1	0.77
	Under diploma		71.7 ± 8.7	
	Diploma		71.7 ± 8.7	
	University		71.5 ± 8.2	

Mother's education level	Illiterate	SBP	117.4 ± 10.3	0.001
	Under diploma		110.3 ± 11.2	
	Diploma		108.5 ± 10.6	
	University		111.7 ± 12.7	
	Illiterate	DBP	74.4 ± 7.8	0.05
	Under diploma		71.3 ± 8.4	
	Diploma		71.4 ± 8.6	
	University		72.7 ± 8.9	

Regular physical exercise	Without physical activity	SBP	109.7 ± 11.7	0.74
	With physical activity		110.3 ± 11.4	
	Without physical activity	DBP	72.3 ± 8.4	0.40
	With physical activity		71.6 ± 8.6	

Participants' education level	The first year of high school	SBP	109.4 ± 10.7	0.50
	The second year of high school		110.1 ± 11.9	
	The third year of high school		110.8 ± 12.1	
	Per university		110.7 ± 10.4	
	The first year of high school	DBP	72.4 ± 8.6	0.02
	The second year of high school		71.5 ± 8.7	
	The third year of high school		70.6 ± 8.5	
	Per university		72.9 ± 8.2	

^*∗*^Systolic blood pressure; ^*∗∗*^diastolic blood pressure; ^*∗∗∗*^data are mean ± SD.
